# Identification of m1A/m6A/m5C/m7G-related genes and clusters associated with neuropathic pain

**DOI:** 10.3389/fneur.2026.1592545

**Published:** 2026-01-29

**Authors:** Liangyuan Tan, Xiaofeng Gan, Dongming Lu, Yingye Liang, Weiyan Fu, Zhengbao Gan, Kailong Wang, Hongliang Tang, Peipei Yang

**Affiliations:** 1 Department of Rehabilitation Medicine, The First Affiliated Hospital of Guangxi University of Traditional Chinese Medicine, Nanning, China; 2 Guangxi Key Laboratory of Molecular Biology of Preventive Medicine of Traditional Chinese Medicine, The First Affiliated Hospital of Guangxi University of Chinese Medicine, Nanning, China; 3 Graduate School, Guangxi University of Chinese Medicine, Nanning, China; 4 Department of Tuina, Fangchenggang Hospital of Traditional Chinese Medicine, Fangchenggang, China

**Keywords:** cluster, differentially expressed gene, neuropathic pain, pathway enrichment, RNA methylation

## Abstract

**Introduction:**

RNA methylation modifications, including N1 methyladenosine (m1A), N6-methyladenosine (m6A), 5-methylcytosine (m5C), and 7-methylguanosine (m7G) methylation, have been increasingly implicated in nervous system disorders. The aim of this study was to explore key m1A/m6A/m5C/m7G-related genes in neuropathic pain (NP).

**Methods:**

NP-related gene expression data were downloaded from a public database. Differentially expressed m1A/m6A/m5C/m7G-related genes between the NP and control samples were screened. Subsequently, the RNA methylation-related clusters of NP were identified. Differentially expressed genes (DEGs) between different clusters were identified; this was followed by functional enrichment, weighted gene co-expression network, and protein–protein interaction analyses. Moreover, m1A/m6A/m5C/m7G-related DEGs were validated in a rat NP model constructed using spinal nerve ligation surgery.

**Results:**

Six m1A/m6A/m5C/m7G-related DEGs were identified between NP and normal samples, namely, *Fto*, *Mettl3*, *Nsun2*, *Ythdf3*, *Wdr4*, and *Eif4e*. Based on these RNA methylation-related genes, two distinct NP clusters were identified. The DEGs between the clusters were involved in multiple pathways, such as the MAPK and FoxO signaling pathways. Among the DEGs, 12, including *Txn1* and *Rps3a*, were identified as key genes. Furthermore, upregulation of *Fto* expression and downregulation of *Mettl3*, *Nsun2*, and *Ythdf3* expression were observed in NP rats compared with those in control rats.

**Discussion:**

Our findings reveal that genes associated with RNA methylation modifications, including *Fto*, *Mettl3*, *Nsun2*, and *Ythdf3*, may be involved in NP progression. Additionally, two RNA methylation-related DEG clusters were identified, and key pathways, such as the MAPK and FoxO signaling pathways, may participate in NP progression.

## 1 Introduction

Neuropathic pain (NP) is a prevalent pain syndrome caused by a lesion or disease of the somatosensory nervous system (1). According to the latest International Association for the Study of Pain (IASP) taxonomy, this chronic pain condition persists or recurs for at least 3 months (2, 3). It encompasses a broad range of peripheral and central disorders, considerably influencing the quality of life of those affected (4). Globally, the prevalence of NP is estimated to be 7–10%, with considerable socioeconomic impact (5, 6). Currently, pain disorders are commonly treated with synthetic drugs such as anticonvulsants, antidepressants, opioids, and serotonin-norepinephrine reuptake inhibitors. Nevertheless, these drugs can have several adverse effects such as addiction, drowsiness, respiratory depression, and cardiovascular complications (7). Additionally, owing to the complex causes and pathological processes, the underlying mechanisms of NP remain largely unclear. Hence, elucidating the key mechanisms of NP may help design more efficient treatments.

RNA methylation is a form of epigenetic regulation involved in RNA translation and degradation. RNA methylation modifications influence various aspects of RNA functionality, including stability, translation, splicing, and intermolecular interactions, thereby serving as crucial mechanisms for post-transcriptional regulation of gene expression. Studies have revealed that RNA methylation is related to various biological processes (8, 9), and its dysregulation plays a key role in the initiation and progression of numerous diseases, including neurological and immune conditions (10). RNA methylation modifications include N1-methyladenosine (m1A), N6-methyladenosine (m6A), N5-methylcytosine (m5C), and N7-methylguanosine (m7G) (11, 12). RNA modifications, particularly the m6A methylation modification, are implicated neural injury and repair, including NP (13). m6A is the predominant methylation modification in eukaryotic RNA, and m6A dysregulation is closely related to ectopic nerve fiber activity in the nervous system, contributing to peripheral and central sensitization (14, 15). This dynamic and reversible modification regulates the expression of pain-associated genes in NP (16). For instance, the methyltransferase METTL3 modulates NP-related neuroinflammation and causes behavioral dysfunction via m6A of SOCS1 (17). METTL14-mediated m6A modification promotes chemotherapy-induced NP through stabilizing GluN2A expression via IGF2BP2 (18). m5C is another widely studied RNA modification that is common in brain tissues and plays a crucial role in cell stress response and apoptosis in neurons (19). The m1A and m7G modifications are also involved in various diseases (20, 21). However, research on NP mainly focuses on m6A modification, and studies related to m1A, m5C, and m7G modifications are limited. The m1A/m6A/m5C/m7G-related genes that are implicated in NP remain largely unknown. Exploration of key m1A/m6A/m5C/m7G-related genes and identification of m1A/m6A/m5C/m7G-related NP subtypes and genes may help predict disease risk and improve the clinical outcomes of NP.

In this study, we downloaded NP-related gene expression data from a public database and acquired information on m1A/m6A/m5C/m7G-related genes from previously published studies (22, 23). We then identified differentially expressed m1A/m6A/m5C/m7G-related genes and validated their expression in a rat NP model constructed using spinal nerve ligation (SNL) surgery. Furthermore, we identified RNA methylation-related clusters and analyzed their related genes and pathways. Overall, we aimed to explore the crucial m1A/m6A/m5C/m7G-related genes and subtypes, shed light on the molecular mechanisms underlying NP, and identify potential therapeutic targets.

## 2 Materials and methods

### 2.1 Data sources and preprocessing

The NP-related gene expression datasets GSE24982, GSE18803, GSE15041, and GSE89224 were downloaded from the Gene Expression Omnibus (GEO) database (24). Due to the limited availability of microarray datasets related to NP and the high degree of homology between rat and mouse genes, the gene datasets from both rats and mice were used for previous studies (25, 26). In this study, we also used datasets from both rats and mice for our bioinformatics analysis. GSE24982 included 20 NP and 20 control L4/L5 dorsal root ganglion (DRG) tissues from adult male *Rattus norvegicus* and was generated on GPL1355 platform. GSE18803 included 6 NP and 6 control ipsilateral dorsal horn tissues from adult *R. norvegicus* and was generated on GPL341 platform. GSE15041 included 10 NP and 6 control L4/L5 DRG tissues from adult/young *R. norvegicus* and was generated on GPL1355 platform. GSE89224 included 33 NP and 7 DRG tissues from adult male *Mus musculus* and was generated on GPL6885 platform. GSE18803 and GSE15041 datasets did not provide the sex information of the rats.

To combine genes from various species, the pruning mean of M-values (TMM) (27) was used to standardize the gene expression data from different datasets. Simultaneously, interspecies gene homologs were selected using the R-package homology. A gene expression matrix with homologous genes from the datasets of different species was compiled. The transcriptome datasets were then merged using Python 3.9 with the rank-in algorithm (28), which could be downloaded at http://www.badd-cao.net/rank-in/index.html (17). Owing to differences in sample processing, measuring equipment, time, and experimental conditions, batch effects were observed in different datasets. To ensure data quality and reliability, the batch effects of different datasets were eliminated using the R SVA package (version 3.52.0) (29).

### 2.2 Acquiring m1A/m6A/m5C/m7G-related genes

The m1A/m5C/m6A-related genes were acquired from a previous study published by Wang et al. (22). The m7G-related genes were obtained from the literature published by Chen et al. (23). The collected m1A/m6A/m5C/m7G-related genes were then transformed into the corresponding rat genes using the R homologene package. After species transformation, 64 rat m1A/m5C/m6A- related genes were identified.

### 2.3 Differential expression analysis

Differentially expressed genes (DEGs) between the NP and control samples were identified using the limma package (version 3.60.6) (30). The cutoff value was set at *p* < 0.05. Additionally, the expression differences in m1A/m6A/m5C/m7G-related genes between NP and normal samples were analyzed to identify m1A/m6A/m5C/m7G-related DEGs.

### 2.4 Correlation analysis

To determine the correlation between the m1A/m6A/m5C/m7G-related DEGs and DEGs, Pearson’s correlation analysis was conducted using the R psych package (version 2.4.6.26) (31). The co-expressed genes were then identified with a threshold value of |*r*| > 0.6 and *p* < 0.05.

### 2.5 Functional enrichment analysis

To understand the biological functions of the co-expressed genes, Gene Ontology (GO) enrichment analysis was performed using the R clusterProfiler package (version 4.12.0) (32). The GO terms included three categories: biological process (BP), cellular component (CC), and molecular function (MF). The enrichment results were considered significant when the *p*-value was <0.05.

### 2.6 Identification of RNA methylation-related clusters

Based on the gene expression matrix of m1A/m6A/m5C/m7G-related DEGs, unsupervised clustering analysis of 69 NP samples was conducted using the R ConsensusClusterPlus package (version 1.68.0) (33). Cluster number (k) was set to 2–5, with hierarchical clustering using the pam algorithm and distance calculated using Spearman’s method. The final cluster number was determined based on the clustering heat map and cumulative distribution plot. Moreover, DEGs between clusters were identified using the limma package (version 3.60.6), with a cutoff value of *p* < 0.05. Furthermore, KEGG pathway and GO term enrichment analyses were conducted using the R clusterProfiler package (version 4.12.0).

### 2.7 Weighted gene co-expression network analysis

Based on the combined dataset, using the identified RNA methylation modification-related clusters as the phenotypic traits, weighted gene co-expression network analysis (WGCNA) was conducted using the R WGCNA package (version 1.73) (34). First, the expression data of DEGs were extracted. To achieve a scale-free topology, network topology analysis was conducted using the pickSoftThreshold function to calculate the soft threshold power and scale-free fitting index (ranging from 1 to 20). When R2 (scale-free fitting index) was greater than 0.9, an appropriate soft threshold was selected. After determining the soft threshold, a topological overlap matrix (TOM) was constructed. The color of each module was divided randomly and the eigenvector of each module was calculated using principal component analysis (PCA). Finally, the correlations between the modules and phenotypes were determined using the module feature vector. Modules with a significant positive correlation with NP were chosen as the key modules, and the genes in the key modules were extracted.

### 2.8 Identification of hub genes

Using the STRING database (35), the protein–protein interactions (PPIs) of key module genes were analyzed. A PPI network was constructed using Cytoscape (version 3.9.0). Hub genes were selected based on the maximum clique centrality (MCC) method using the cytoHubba (36) plug-in in Cytoscape.

### 2.9 Animals

Male specific-pathogen-free (SPF) Sprague–Dawley rats weighing 200 g were purchased from Changsha Tianqin Biotechnology Co., Ltd. (Production License No.: SCXK2022-0011). These rats were housed at the Scientific Experiment Center of Guangxi University of Chinese Medicine (Use License No.: SYXK2019-0001) under controlled conditions (12-:12-h light/dark cycle, temperature 20 °C–25 °C), with free access to food and water. Cages and bedding were changed daily. After a 7-day acclimation period, the rats were used in the experiments. This study utilized sample blinding and incorporated independent biological replicates for each time point to mitigate individual variability. This study was approved by the Animal Research Ethics Committee of Guangxi University of Chinese Medicine (No. DW20240319-057). The protocol strictly complied with the Guidelines for the Management and Use of Experimental Animals (2011, 8th Edition) and the relevant experimental regulations formulated by the Animal Testing Department of the University.

### 2.10 SNL surgery for constructing an NP model

The rats were anesthetized using an intraperitoneal injection of pentobarbital sodium (40 mg/kg). After anesthesia, the rats were placed in prone position and securely fixed on the operating table. Following thorough skin cleaning and disinfection with alcohol, an incision approximately 2 cm in length was made longitudinally on the left side of the L4–S1 spinous processes. The skin and subcutaneous tissue were then incised and blunt dissection was performed to separate the left paraspinal fascia and muscles, exposing the transverse process of the L6 vertebra. The L6 transverse process was completely removed using a pair of bone forceps to expose the L5 spinal nerves. The left L5 spinal nerve was isolated and tightly ligated with 6–0 silk sutures distal to the dorsal root ganglion to ensure that no excessive tension was applied to the nerve during the procedure. After hemostasis and cleaning, the wound was closed layer-by-layer, and an erythromycin ointment was applied to prevent infection. Post-surgery, the rats were kept warm and provided adequate food and water.

### 2.11 Mechanical allodynia (von Frey test)

Mechanical allodynia was assessed based on the paw withdrawal threshold (PWT) value in response to von Frey filament stimulation. This test was performed once daily for 2 days before modeling, and the average value was taken as the baseline value. After model establishment, the PWT test was performed on days 3, 7, and 14 to observe changes in mechanical PWT. The rats were placed in a transparent plastic container with a wire-mesh bottom. When the rats adapted to the surrounding environment, an electronic Plantar Mechanical Nociception Device (KW-CT-1; Nanjing Calvin Biotechnology Co., Ltd., Nanjing, China) was used to apply a vertical stimulus to the center of the rats’ left hind paws through the wire mesh at the bottom. When the rats exhibited positive behaviors, such as licking and withdrawing their paws, the stimulus was stopped, and the data were recorded. After performing the measurements three times, with a 5-min interval between tests, the average value was considered the PWT value of the rats.

### 2.12 Thermal hyperalgesia (hot plate test)

Thermal hyperalgesia was evaluated based on the paw withdrawal latency (PWL) value. Before model establishment, the PWL test was conducted once daily for 2 days, and the average value was taken as the baseline value. After model establishment, the PWL test was performed on days 3, 7, and 14 to observe changes in the PWL. The room temperature was 25 °C. Once the temperature of the paw analgesic apparatus (KW-RB; Nanjing Calvin Biotechnology Co., Ltd.) stabilized at 50 °C, each rat was placed in a closed, transparent glass box, and the timer was started. When rapid lifting or frequent licking of the paw was observed, the timer was stopped, and the time was recorded. This procedure was performed three times, with a 5-min gap between each trial, and the average value was considered the PWL value of the rats.

### 2.13 Real-time quantitative PCR

Total RNA was extracted from the L4-L6 spinal cord tissues using the TriQuick reagent (R1100, Solarbio, Beijing, China). Reverse transcription of the RNA to cDNA was performed using HyperScript III RT SuperMix for qPCR with gDNA Remover (R202-02, Xinbei (Shanghai) Biotechnology Co., Ltd., Shanghai, China). qPCR was performed using the 2 × S6 Universal SYBR qPCR Mix (Q204-01, Xinbei (Shanghai) Biotechnology Co., Ltd.). *GAPDH* was used as the internal reference. Finally, the relative expression levels of *Nsun2*, *Mettl3*, *Ythdf3*, *FTO*, *Wdr4*, and *Eif4e* were calculated using the 2^−ΔΔCt^ method. Primers used in this assay are listed in Table 1.

**Table 1:** Primers for qPCR.

Gene	Primers	Sequences (5′-3′)	Product length
Fto	Forward	GACGAGTTCTATCAGCAGTGGC	138 bp
	Reverse	GTCCCGAAACAAGCAGCCAT	
Mettl3	Forward	GCAGCGCATCATCAGGACAG	147 bp
	Reverse	TGACTGGTGGAACGAACCTCA	
Nsun2	Forward	AGTTCCTCAGCCACTAAGCTG	139 bp
	Reverse	GCTGATGTTTCCAGACTCCGT	
Ythdf3	Forward	GGGCAAGGAAATAAAGTTTCAGT	131 bp
	Reverse	GGATCTGACATTGGTGGATAGC	
Wdr4	Forward	TGACCAGTTTGTGCTCACCG	111 bp
	Reverse	CGGCTCACAAACTCCGTGTG	
Eif4e	Forward	CAGGAGGTTGCTAATCCAGAGC	117 bp
	Reverse	AGAGATCAACCGAAGGTTTGCT	
GAPDH	Forward	TCTCTGCTCCTCCCTGTTCT	95 bp
	Reverse	ATCCGTTCACACCGACCTTC	

### 2.14 Western blot assay

After euthanizing the rats with overdose anesthesia, the L4–L6 spinal cord tissue was quickly harvested on ice. Total protein was extracted using a pre-chilled RIPA lysis buffer containing PMSF. After determining protein concentration using the BCA method (Yamei), 10% SDS-PAGE was carried out for protein separation. The proteins were transferred onto polyvinylidene fluoride (PVDF) membranes (Millipore, United States). After blocking, the membranes were incubated with the following primary antibodies: Nsun2 (1:1000, PH6626, ab-mart, Shanghai, China), Mettl3 (1:1000, 15,073-1-AP, Proteintech, Rosemont, IL, United States), Ythdf3 (1:500, 25,537-1-AP, Proteintech), FTO (1:1000, PA2776, ab-mart); Wdr4 (1:1000, PS17092, ab-mart), Eif4e (1:1000, 11,149-1-AP, Proteintech), and GAPDH (1:20000, 10,494-1-AP, Proteintech) overnight at 4 °C. The next day, after washing the membranes thrice with Tris-buffered saline with Tween 20 (TBST), a secondary antibody (horseradish peroxidase (HRP) goat anti-rabbit IgG, 1:5000, SA00001-2, Proteintech) was added, and the membranes were incubated for 1 h. After rinsing the membranes with TBST, the proteins were detected using ECL reagent. The band intensity was quantified using ImageJ software. GAPDH was used as an internal control.

### 2.15 Statistical analysis

Experimental data are presented as mean ± standard deviation (SD). Statistical analyses were performed using GraphPad Prism 8 (GraphPad Software, United States), and results with a *p*-value of <0.05 were considered statistically significant.

## 3 Results

### 3.1 DEG screening

**Figure 1:**
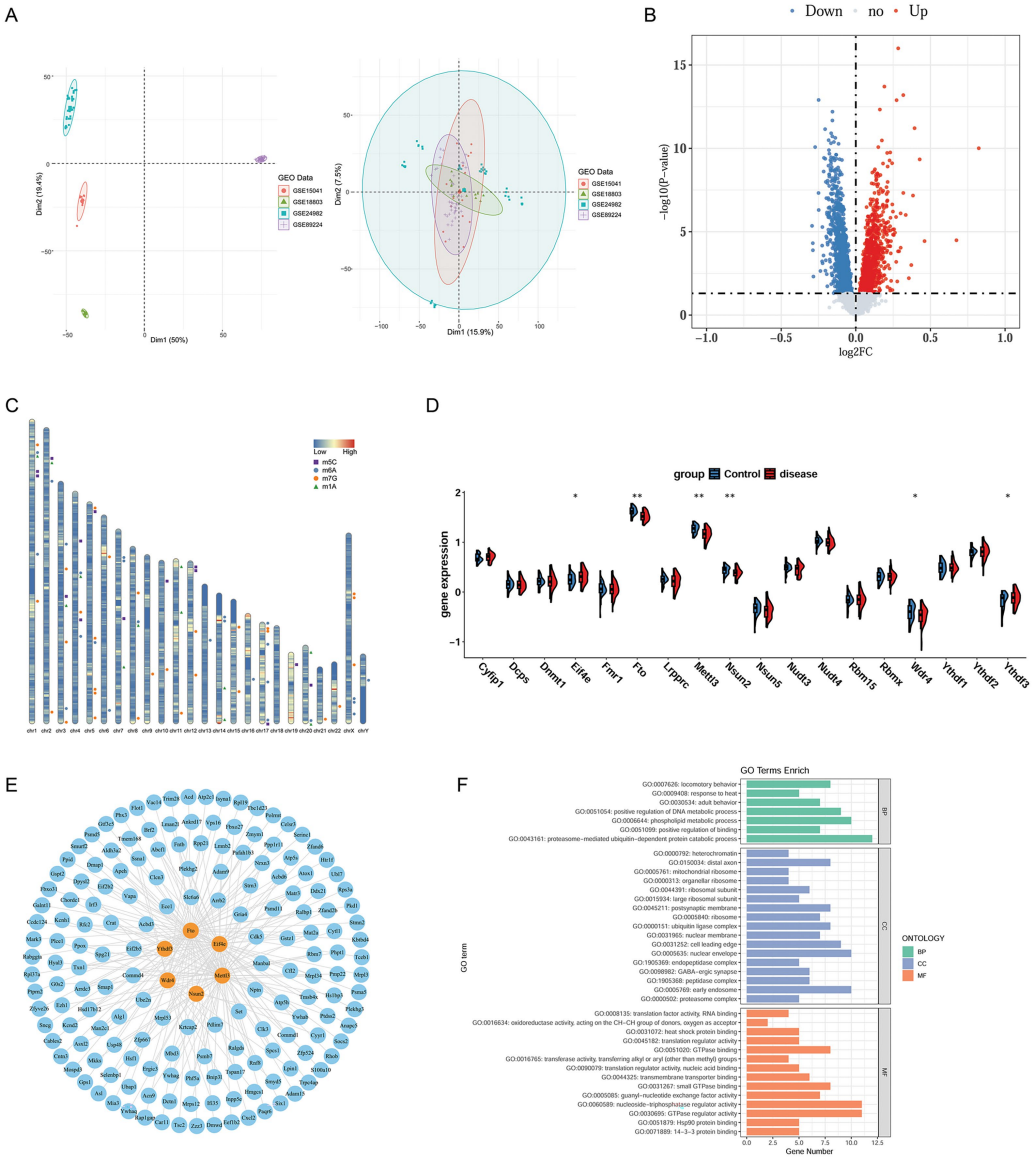
Data preprocessing and identification of m1A/m6A/m5C/m7G-related DEGs. **(A)** PCA of samples from the four datasets before and after batch effect removal. **(B)** Volcano plot of DEGs between NP and control samples. The red nodes indicate upregulated genes and blue nodes indicate downregulated genes. **(C)** Chromosomal distribution map of m1A/m6A/m5C/m7G-related genes. In the gene chromosome distribution map, the shading indicates gene density within a specific chromosome region. Darker areas represent regions with higher gene density, indicating that they contain more genes, whereas lighter areas indicate lower gene density with fewer genes. **(D)** Box plot of the 18 m1A/m6A/m5C/m7G-related genes. *p < 0.05, **p < 0.01, ***p < 0.001, and ****p < 0.0001. **(E)** Correlation network of the six m1A/m6A/m5C/m7G-related DEGs and 2,416 DEGs. The blue nodes are DEGs with a correlation coefficient greater than 0.6 and *p* < 0.05. The yellow nodes represent the six m1A/m6A/m5C/m7G-related genes. **(F)** GO enrichment results. NP, neuropathic pain; DEGs, differentially expressed genes; PCA, principal component analysis; m1A, N1 methyladenosine; m6A, N6-methyladenosine; m5C, 5-methylcytosine; m7G, 7-methylguanosine; GO, gene ontology.

Through data preprocessing, the batch effects of the four datasets (GSE24982, GSE18803, GSE15041, and GSE89224) were successfully eliminated, and the datasets were combined (Figure 1A). In total, 2,416 DEGs were detected between NP and normal samples, with 987 upregulated and 1,429 downregulated (Figure 1B).

### 3.2 Identification of m1A/m6A/m5C/m7G-related DEGs

The m1A/m6A/m5C/m7G-related genes obtained from the literature were sorted. After removing the duplicates, 76 genes were obtained. To present the specific locations of these genes on the chromosomes more objectively, a chromosome distribution map for these genes was drawn (Figure 1C). Through further species transformation, these m1A/m6A/m5C/m7G-related genes were transformed into 64 rat genes. Among these genes, only 18 were expressed in the combined dataset associated with NP. The differential expression analysis revealed that six genes were differentially expressed between NP and normal samples: *Fto*, *Mettl3*, *Nsun2*, *Ythdf3*, *Wdr4*, and *Eif4e* (Figure 1D).

### 3.3 Correlation analysis

The Pearson correlation between the six m1A/m6A/m5C/m7G-related DEGs and 2,416 DEGs was analyzed, obtaining 168 co-expressed genes. The correlation network is shown in Figure 1E.

### 3.4 Functional enrichment analysis

The GO term enrichment analysis was conducted to reveal the biological functions of the co-expressed genes. A total of 38 GO terms were significantly enriched by the co-expressed genes, including seven BP terms such as proteasome-mediated ubiquitin-dependent protein catabolic process, 17 GG terms such as nuclear envelope, and 14 MF terms such as GTPase regulator activity (Figure 1F).

### 3.5 Identification of RNA methylation-related clusters

**Figure 2:**
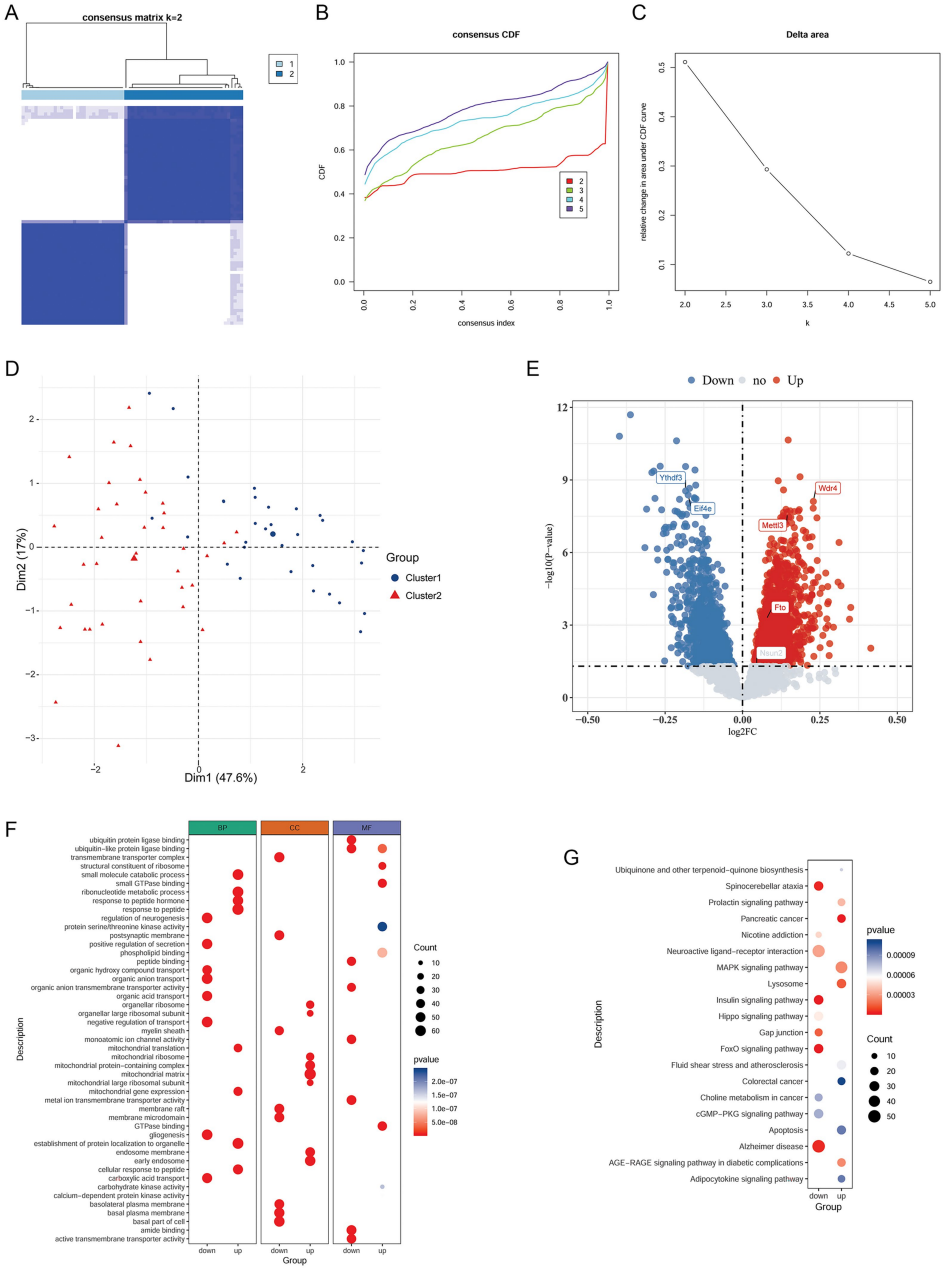
Identification of NP-related clusters and analysis of cluster-related genes and pathways. **(A)** Heatmap of the clusters. **(B)** CDF distribution curve. **(C)** Delta-area line graph. **(D)** PCA plot of sample distribution in the two clusters. **(E)** Volcano plot of DEGs between the clusters. **(F)** GO enrichment results. **(G)** KEGG enrichment results. NP, Neuropathic pain; CDF, cumulative distribution function; PCA, principal component analysis; DEGs, differentially expressed genes; KEGG, Kyoto Encyclopedia of Genes and Genomes.

Based on the expression data of the six m1A/m6A/m5C/m7G-related DEGs, 69 NP samples were grouped into two clusters using unsupervised clustering analysis (Figures 2A–C). Cluster1 contained 32 samples, and Cluster2 contained 37 samples. The PCA showed that samples within the same cluster clustered together, and the two clusters were clearly separated (Figure 2D), indicating the reliability of the analysis.

### 3.6 Differential analysis of clusters and functional enrichment analysis

To better understand the differences between the clusters, we analyzed the DEGs between the clusters. In total, 2,514 DEGs (1,415 upregulated and 1,009 downregulated) were identified between the clusters (Figure 2E). Among the six previously identified m1A/m6A/m5C/m7G modification regulators, the expression of *Mettl3*, *Wdr4*, and *Fto* was upregulated in the cluster 1 compared to cluster 2, whereas that of *Ythdf3* and *Eif4e* was downregulated in the cluster 1. However, *Nsun2* showed no significant difference between the clusters (Figure 2E). Subsequently, a functional enrichment analysis was performed. The upregulated genes between the clusters were significantly involved in 1589 GO BP terms, 202 GO CC terms, and 220 GO MF terms, and the downregulated genes were markedly involved in 2074 GO BP terms, 219 GO CC terms, and 309 GO MF terms. The top eight GO terms in each category are shown in Figure 2F. Moreover, the upregulated and downregulated genes were found to be involved in 57 KEGG pathways including the MAPK signaling pathway and 65 KEGG pathways including the FoxO signaling pathway, respectively. The top 20 KEGG pathways are shown in Figure 2G.

### 3.7 Analysis of clinically significant modules and module genes

**Figure 3:**
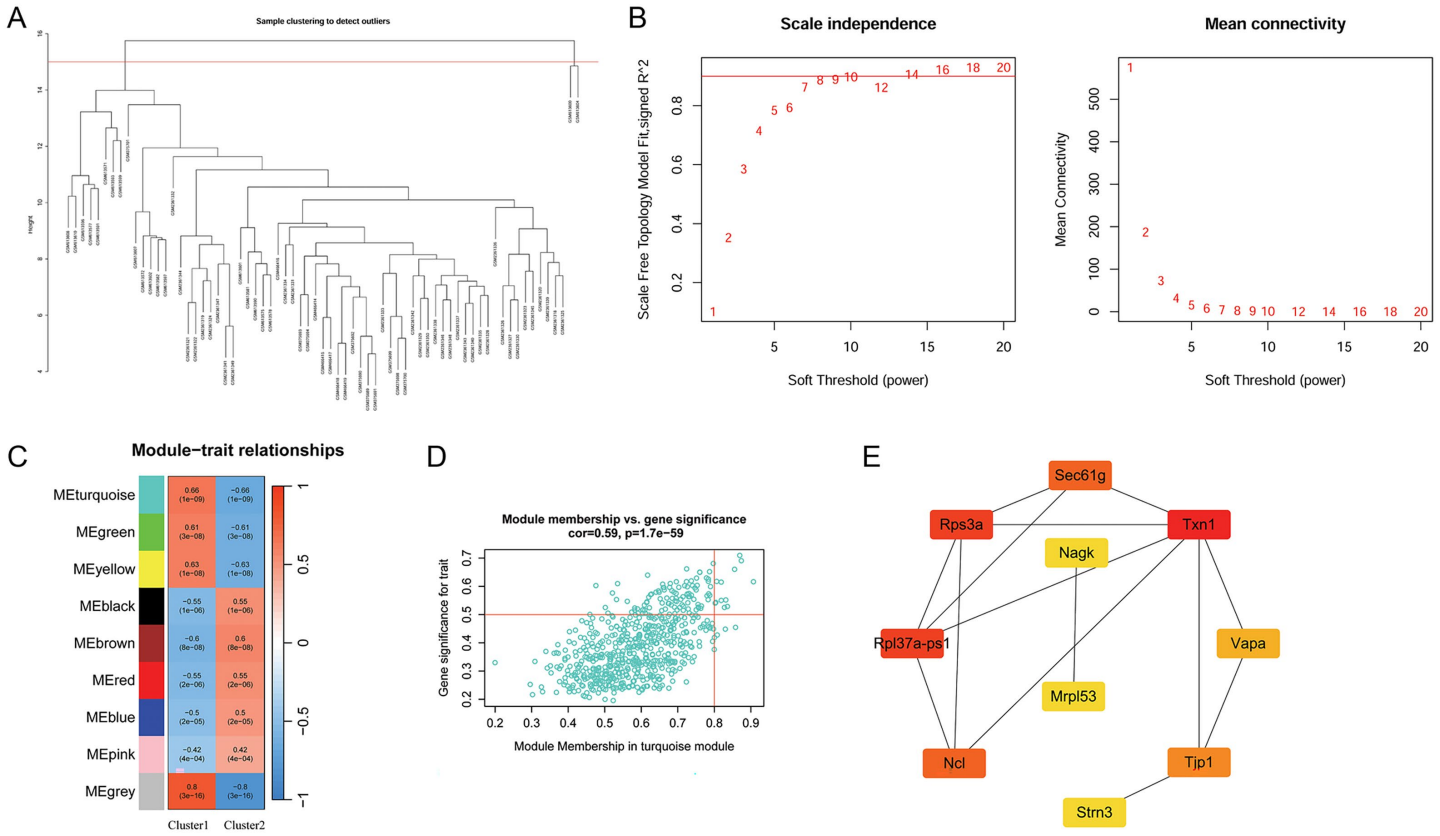
Analysis of clinically significant modules using WGCNA and hub gene identification. **(A)** Analysis of outlier samples (GSM613600 and GSM613604). **(B)** Scale-free soft-threshold distribution. **(C)** Heat map of the correlation between modules and clusters. **(D)** Scatter plot of module membership in the MEturquoise module and gene significance for NP. **(E)** PPI network of the top 10 genes identified using the cytoHubba plug-in. The darker the color of the node, the higher the importance score in the network. WGCNA, weighted gene co-expression network analysis; NP, neuropathic pain; PPI, protein–protein interaction.

The WGCNA was performed to identify clinically significant modules associated with RNA methylation modification-related clusters. After sample clustering, the outlier samples GSM613600 and GSM613604 were removed (Figure 3A). Based on R2 (scale-free fit index) greater than 0.9, the soft threshold was determined to be a power of seven to obtain a scale-free network (Figure 3B). Subsequently, nine modules were obtained, and Pearson’s correlation analysis was used to analyze the association between each module and cluster. A heat map of module–trait relationships is shown in Figure 3C. Among these modules, MEturquoise had the highest association with the clusters (|*r*| = 0.66, *p* < 0.05) and was considered a key module associated with the clusters. A total of 621 genes in this module were extracted for subsequent analysis.

### 3.8 Hub gene selection

Among the 621 genes in the MEturquoise module, 12 were selected as key genes in this module with the cutoff value of the module membership of a gene of >0.8 and gene significance of >0.5 (Figure 3D). Subsequently, a PPI network was constructed using these 12 genes. Using the cytoHubba plug-in, the top 10 genes were selected. Among these 10 genes, Txn1 and Rps3a had the highest MCC scores. The PPI network established using the top 10 genes is shown in Figure 3E.

### 3.9 Validation of the six m1A/m6A/m5C/m7G-related DEGs in the rat NP model

**Figure 4:**
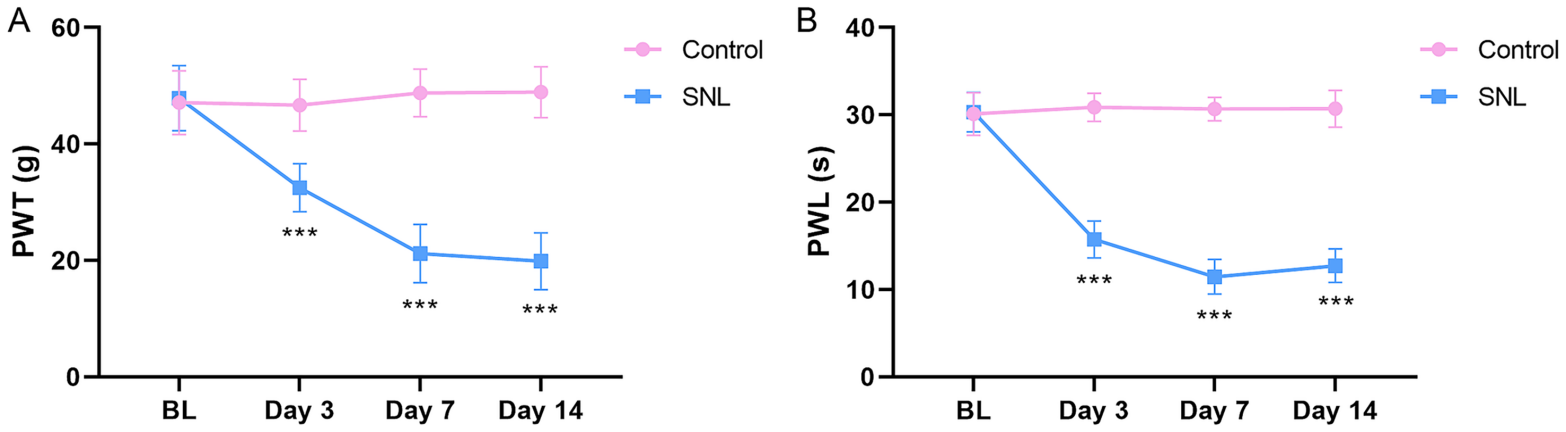
Paw withdrawal threshold **(A)** and paw withdrawal latency **(B)** of rats 3, 7, and 14 days after SNL surgery. *n* = 6 per group. SNL, spinal nerve ligation. ***p < 0.001.

**Figure 5:**
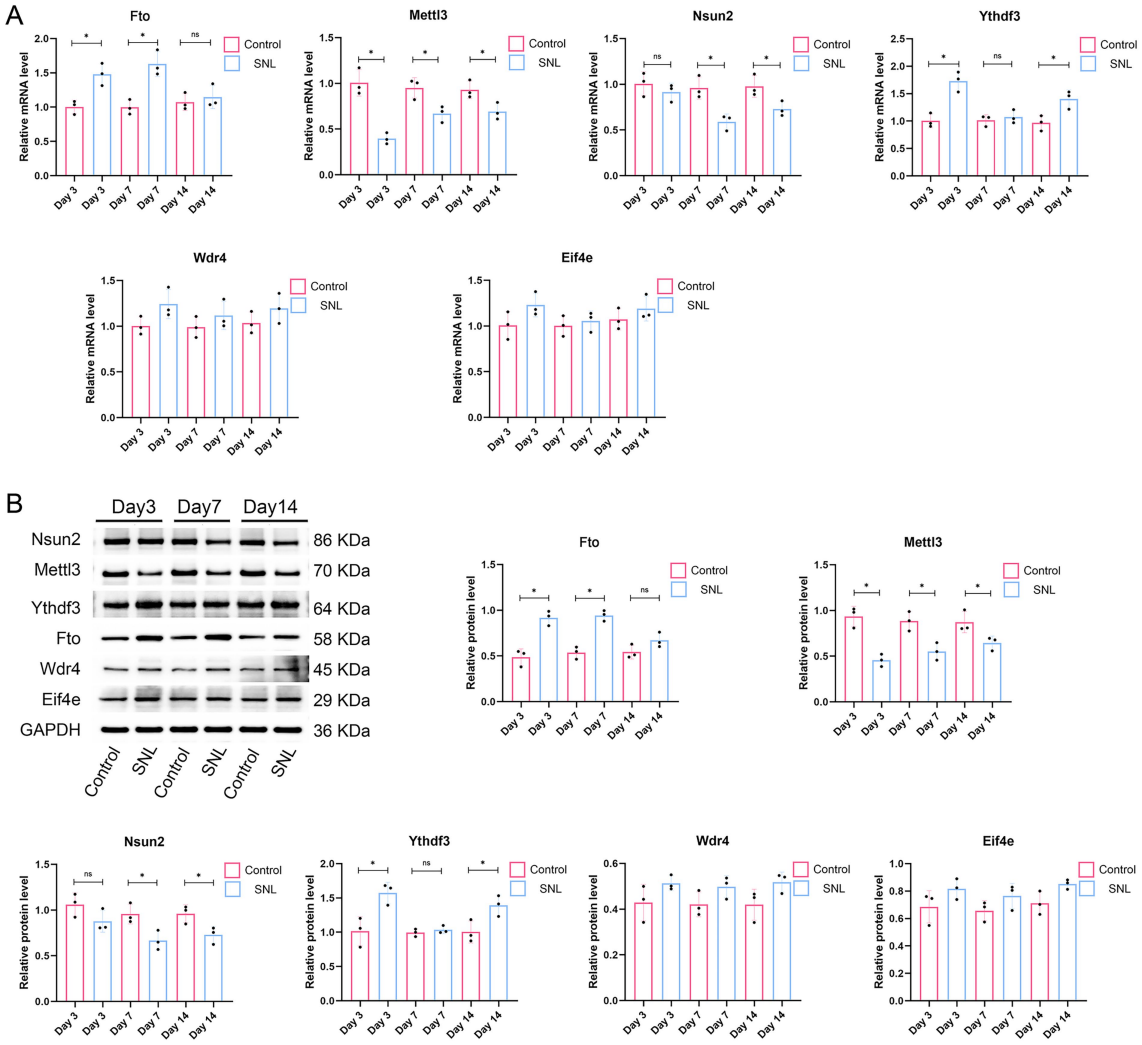
Validation of expression of the six m1A/m6A/m5C/m7G-related DEGs (*FTO*, *METTL3*, *NSUN2*, *YTHDF3*, *WDR4*, and *EIF4E*) in rats after 3, 7, and 14 days of SNL surgery. *n* = 3 per group. SNL, spinal nerve ligation. **(A)** qPCR results. **(B)** Western blot analysis results. *p < 0.05.

To investigate whether the six m1A/m6A/m5C/m7G-related DEGs are involved in NP, an SNL model was established to simulate NP. We evaluated pain response using PWL and PWT metrics. The results showed that the PWT and PWL metrics were significantly reduced on days 3, 7, and 14 post-surgery in the SNL group compared with those in the control group (*p* < 0.001; Figure 4), indicating the successful establishment of an SNL model. We then investigated the expression of the six m1A/m6A/m5C/m7G-related genes (*Fto*, *Mettl3*, *Nsun2*, *Ythdf3*, *Wdr4*, and *Eif4e*) in the SNL and control groups. The qPCR results indicated a significant increase in *Fto* expression in the SNL group on days 3 and 7 post-surgery compared with that in the control group. Additionally, downregulation of *Mettl3* expression was observed in the SNL group on days 3, 7, and 14 post-surgery (*p* < 0.05). Downregulation of *Nsun2* expression was noted in the SNL group on days 7 and 14 post-surgery, and reduced *Ythdf3* expression was observed in the SNL group on days 3 and 14 post-surgery (*p* < 0.05). However, *Wdr4* and *Eif4e* expression levels were not significantly different between the groups (Figure 5A). Consistent changes in the expression of these proteins in the SNL group across these intervals were observed using western blotting (Figure 5B).

## 4 Discussion

The NP that occurs after nerve damage is often refractory (37). Various mechanisms underlying NP development have been reported (18, 38), but many patients continue to experience unresolved pain, indicating that our understanding of its origin remains incomplete (39). In this study, we identified six RNA methylation-related genes involved in NP. Among them, *Fto*, *Mettl3*, *Nsun2*, and *Ythdf3* were differentially expressed in the rat NP model. Additionally, based on these RNA methylation-related genes, we identified two NP clusters and analyzed DEGs and their pathways between the clusters, which may be of significance for developing individualized treatment strategies for NP.

Epigenetic regulation at the gene level has garnered attention with increasing research on NP (40). RNA methylation modification is a reversible and dynamically controlled process involved in multiple biological processes (41). RNA methylation modifications are emerging as key regulators of the nervous system (42). Emerging research has underscored the significance of m6A methylation in the context of nerve injury, whereas the roles of m1A, m5C, and m7G modifications in RNA have opened up new directions for exploration (13). To investigate the possible mechanism underlying RNA methylation modifications in NP, we analyzed and identified six m1A/m6A/m5C/m7G-related DEGs in NP. Fto functions as a demethylase to facilitate the m6A modification of mRNA, which has been implicated in both the initiation and maintenance of NP (43). Research has revealed that Fto-mediated m6A modification of G9a contributes to the manifestation of NP symptoms (44). In contrast, Fto downregulation has been observed to relieve NP by suppressing oxidative stress through the downregulation of GRP177 expression (45). Additionally, the knockdown of *Fto* alleviates NP progression by enhancing CXCR3 methylation (46). Mettl3 is an m6A methyltransferase that facilitates the catalysis of m6A modifications on mRNA. A previous study has revealed that Mettl3 modulates NP development via m6A methylation of *SOCS1* (17). Mettl3 can inhibit NP development through N6-methyladenosine-dependent primary miR-150 processing (47). Ji et al. (48) have reported that esketamine mitigated depressive-like behaviors in NP mice by modulating the Mettl3–GluA1 pathway, suggesting that METTL3 may be a promising therapeutic target for NP. Nsun2 is a methyltransferase that catalyzes the m5C modification of multiple types of RNAs, including mRNAs and non-coding RNAs (49). In the DRG, Nsun2 modulates acute sensory hypersensitivity in response to peripheral injury (50). Ythdf3 is a major “reader” protein that can identify m6A nucleotides through its YTH domain (51). It is overexpressed after chronic spinal cord injury and is predominantly expressed in neurons (52), suggesting its potential involvement in neuroplastic changes. Wdr4 acts as a critical partner of Mettl3 and is indispensable for m7G modification of tRNA (21). Wdr4 has been reported as a crucial contributor to neurodevelopmental disorders (53). Eif4e modulates the process of translation initiation by binding to an mRNA “cap” structure and is involved in the sensitization of pain circuits (54). Eif4e phosphorylation is a major regulator of nociceptive plasticity and has been implicated in the progression of chronic pain (55). In the present study, we confirmed the differential expression of *Fto*, *Mettl3*, *Nsun2*, and *Ythdf3* in NP rats, suggesting that these genes may be crucial contributors in the development of NP. However, *Wdr4* and *Eif4e* did not show significant differences in expression in NP rats. Further investigation is required to determine whether Wdr4 and Eif4e are involved in NP development. Overall, these findings underscore the importance of understanding how RNA modification-related genes contribute to the complex mechanisms of NP, which can inform the development of targeted therapies and enhance our knowledge of pain biology.

Research suggests that different gene expression patterns contribute to unique neurochemical, physiological, and functional characteristics of DRG neuron subtypes, with each subtype or cluster potentially contributing differently to nerve regeneration and pain (16). The identification of different NP clusters may help identify novel target genes and develop promising therapies to optimize NP treatment. Therefore, we further identified two distinct NP clusters based on the six m1A/m6A/m5C/m7G-related DEGs. By correlating specific gene expression patterns with clinical outcomes, it is possible to more accurately predict the progression of diseases and the response to treatment, thereby ultimately enhancing the therapeutic effect for patients. To gain a deeper understanding, we screened the key DEGs between the clusters, such as *Txn1* and *Rps3a*, providing novel insights into the pathophysiology of NP. Additionally, we found that the DEGs between the clusters were involved in pathways such as the MAPK and FoxO signaling pathways. MAPKs participate in multiple aspects of cell signaling and are essential for modulating nociceptive information, along with both peripheral and central sensitization induced by intense noxious stimuli (56). Sinomenine can reduce inflammation in the DRG to suppress NP development through the p38 MAPK/CREB signaling pathway (57). The FoxO signaling pathway participates in cell proliferation and apoptosis (58). Previous research has indicated that the FoxO signaling pathway may be triggered in uninjured L4 DRG in an L5 SNL model, offering potential mechanisms for SNL-induced NP (59). Therefore, we speculated that these pathways may contribute to the development and progression of NP. The insights gained from the analysis of these key DEGs and associated pathways not only enhance our understanding of the molecular mechanisms underlying NP but also open avenues for future research aimed at developing targeted therapies that can effectively modulate these signaling pathways to improve patient outcomes.

This study had some limitations. First, the functions of RNA methylation-related genes in NP development were not validated using functional experiments. Incorporating functional studies, such as knockdown or overexpression of specific RNA methylation-related genes, to test whether modulating these epigenetic changes truly alters pain-like behaviors in animals in this model would provide critical insights into the biological mechanisms underlying NP. Second, the involvement of key pathways such as the MAPK and FoxO signaling pathways in NP progression was not experimentally investigated. Third, the clinical application of the two identified clusters for patient stratification was not verified in clinical cohorts. Further research is required to validate our findings. Finally, the animal experiments in this study were conducted exclusively on male rats. Given the sex differences in pain perception and mechanisms, the conclusions of this research primarily apply to male SD rats. Future parallel experiments in female animals are crucial for validating the universality of our findings and exploring potential sex-specific regulatory pathways.

In conclusion, key RNA methylation-related genes, particularly *Fto*, *Mettl3*, *Nsun2*, and *Ythdf3*, may be involved in the progression of NP. Moreover, two RNA methylation-related NP clusters were identified, and key pathways, such as the MAPK and FoxO signaling pathways, may contribute to NP progression. These findings provide a foundation for the development of personalized treatments for NP.

## Data Availability

The datasets presented in this study can be found in online repositories. The names of the repository/repositories and accession number(s) can be found in the article/supplementary material.
